# Concurrent emergencies: overlapping *Salmonella* and COVID-19 concerns in public health strategies and preparedness

**DOI:** 10.3389/fpubh.2024.1331052

**Published:** 2024-04-29

**Authors:** Windra Prayoga

**Affiliations:** Department of Biology, Faculty of Biotechnology, University of Surabaya, Surabaya, Indonesia

**Keywords:** co-infections, epidemiological connections, foodborne disease in pandemic, pathogens, public health implication, SARS-CoV-2

## Introduction

The COVID-19 pandemic has prompted a significant scholarly investigation into its ramifications, yet there is a further menace from *Salmonella* (1). *Salmonella* is a prominent public health concern worldwide, particularly in countries with limited resources (2). The remarkable adaptability of this organism to various conditions demonstrates genetic and phenotypic changes that enhance its ability to survive and reproduce (3), allowing it to thrive under diverse environmental conditions. *Salmonella* survives in different habitats and overcomes barriers that affect how the disease develops, its severity, and how it interacts with the host. It leads to the emergence of *Salmonella* strains with the remarkable capacity to induce a range of health problems (4).

*Salmonella* infections remain a growing issue, but with less acknowledgment owing to the prevailing emphasis on the COVID-19 pandemic in the last 3 years. As a result of the COVID-19 pandemic, healthcare has redirected its focus toward detecting the SARS-CoV-2 virus, primarily in clinical settings. These alterations in clinical diagnostic emphasis, therefore, exclude the possibility of diagnosing other infectious disorders, such as those caused by *Salmonella* (5). The widespread enforcement of strict lockdown measures worldwide during the COVID-19 pandemic restricted individual and societal activities. Moreover, it also impacted the distribution of agricultural commodities, livestock, and food products, ultimately causing disruptions in food supply chains (6, 7).

While the lockdown may have contributed to a decrease in the likelihood of *Salmonella* infection, it is important to note that *Salmonella* still persists (8). Although *Salmonella* infection and COVID-19 have different modes of transmission, they exhibit comparable symptoms (9). The misreading of this circumstance, along with the shift in diagnostic focus, amplifies the bias in estimating the incidence of *Salmonella* infections (5). Additionally, it is essential to note that the immunological response that SARS-CoV-2 elicits may have affected the severity of bacterial infections like *Salmonella* (10). These factors were later identified to have severe implications for the occurrence and management of *Salmonella* infections (4, 11, 12).

The interplay between these pathogens exhibits a multifaceted nature. The persistence of *Salmonella* during the COVID-19 pandemic (8, 13) necessitates the attention of individuals in academia, healthcare professions, and policy-making positions. Despite the inherent disparities between these pathogens, they exhibit a notable interconnection (8, 14). There have been documented instances of co-infection between *Salmonella* and COVID-19 in the nine studies worldwide (15), some of them were found in patients from Pakistan (13), Turkey (1), and Japan (14). Patients who have a co-infection face more severe repercussions and increase the complexity of therapy solutions needed to address this issue. Furthermore, incorrect therapeutic intervention might exacerbate the problem and perhaps result in greater mortality rates (1).

This article examines the dynamic relationship between *Salmonella* and COVID-19, employing an evaluation of empirical evidence to analyze their intersections across clinical and social domains. The primary objective of this initiative is to offer a complete comprehension of the further framework of infectious diseases, therefore providing valuable insights into the development of methods for effectively addressing different health emergencies. In extraordinary circumstances, it is imperative to recognize the necessity of adopting a comprehensive strategy to address the challenges posed by contagious diseases. Understanding the interplay between infections such as *Salmonella* and SARS-CoV-2 enables the advancement of more effective strategies.

## *Salmonella* during COVID-19: overlapping symptoms and diagnostic challenges

The many serotypes of *Salmonella*, all of which are regarded as pathogens, can be attributed to variations in their virulence mechanisms, the intensity of the resulting sickness, and their ability to adapt to different hosts (16, 17). Consequently, this bacterium causes a diverse array of infections (18, 19), ranging from mild gastroenteritis to more severe systemic disorders including bacteremia and typhoid fever (4, 20). A *Salmonella* infection commonly manifests gastrointestinal symptoms such as abdominal pain, nausea, constipation, and diarrhea. At times, the condition may present itself in a more severe form, leading to issues such as dehydration and imbalances in electrolyte levels (3).

Occasionally, infection with non-typhoidal *Salmonella* can lead to septicemia, arthralgia, and other complications. Nevertheless, in the event of typhoid fever, symptoms often manifest within a span of 1–3 weeks following exposure to the bacteria. Initial symptoms include a low-grade fever that progressively intensifies over the day, perhaps peaking at 104 degrees Fahrenheit (40°C) (2, 21). Other indications, such as chills, headaches, debilitation, muscular discomfort, abdominal pain, and gastrointestinal disturbances such as diarrhea or constipation, still exist (16). Individuals afflicted with typhoid fever may further experience symptoms such as a respiratory cough, diminished desire for food, and perspiration (2, 22). *Salmonella* is commonly spread by the fecal-oral route, which implies that infection with this bacterium occurs when contaminated foods or beverages are consumed. The extent of *Salmonella* transmission is influenced by the particular serovar and the circumstances of transmission (23).

On the other hand, COVID-19 is predominantly transmitted by respiratory droplets and aerosols, which can be inhaled or encounter mucosal membranes. Its transmission typically varies based on factors such as the existence of asymptomatic persons, the efficacy of public health efforts, and the population density (24). The SARS-CoV-2 virus, which is the cause of COVID-19 disease, exhibits a wide range of symptoms that can vary in intensity. The typical symptoms include a high body temperature, coughing, and difficulty breathing. Tiredness and physical discomfort are frequently present as well. Additionally, certain individuals may have symptoms such as headaches, an aching throat, a diminished sense of taste or smell, and gastrointestinal issues, including nausea or diarrhea. It is crucial to acknowledge that the manifestation of COVID-19 symptoms can vary significantly across individuals, with some being asymptomatic carriers and others experiencing severe respiratory distress (25).

Identifying patients based on their symptoms is a prevalent diagnostic strategy in most clinical settings. Nevertheless, the symptoms associated with the SARS-CoV-2 virus, such as fever, headache, reduced appetite, muscular discomfort, and gastroenteritis (nausea and diarrhea), have a remarkable resemblance to those observed in *Salmonella* infections, including both typhoid and non-typhoid strains (9). This recurring overlap of symptoms between these pathogens during the COVID-19 pandemic made it challenging to accurately identify *Salmonella*, giving rise to diagnostic bias in clinical settings (5). Additionally, according to Ürkmez and Atalay (1) as well as Haqqi et al. (13), the simultaneous occurrence of COVID-19 and *Salmonella* infections presents significant challenges in the planning of clinical treatment (8, 26). It is crucial to acknowledge that although these symptoms may be similar, COVID-19 is mostly a pulmonary disease, while *Salmonella* infection usually manifests with gastrointestinal infections. Therefore, these medical conditions require specific therapeutic interventions that are efficient against their pathogens (9).

## Potential approaches for diagnosis strategies

Given the above concerns, it is essential to utilize diagnostic approaches that exhibit both adaptability and accuracy in addressing these problems. Utilizing precise and reliable diagnostic techniques is crucial for making succinct decisions while dealing with these overlapping symptoms. Accurate identification of pathogens implicated in illnesses is crucial for effective treatment (5, 14). Conversely, inadequate treatment might exacerbate the condition for both the patient and the whole scenario. The implementation of comprehensive solutions is essential for the efficient management of both COVID-19 pandemics and *Salmonella* threats (8).

Thus, a clinical diagnosis based on symptoms should be supplemented with a prompt laboratory diagnosis, particularly using simple and rapid molecular methods to provide more precise results. Due to its high efficiency and accuracy, molecular approaches are strongly recommended over serology techniques for identifying *Salmonella* and SARS-CoV-2, as the latter has been found to provide misleading findings (27). Several well-established molecular approaches for *Salmonella* and SARS-CoV-2 identification are extremely specific and precise. However, the use of Real-Time Polymerase Chain Reaction (qPCR) for detection is particularly effective and suitable for routine diagnosis (28). Although Next Generation Sequencing (NGS) can serve as a more thorough method for identification due to its ability to provide a full genomic study, it is much more costly compared to qPCR (29). Once the specific infectious microorganisms, such as *Salmonella* or SARS-CoV-2, have been definitively identified, the proper therapeutic measures may be taken. This includes administering antibiotics to treat *Salmonella* and antiviral medications to treat SARS-CoV-2 (28).

## Shifting priorities: COVID-19's impact on healthcare and food safety

The global COVID-19 pandemic has led to a substantial restructuring of healthcare priorities and the distribution of resources (30, 31). The healthcare system had significant difficulty in properly treating numerous health concerns due to the new coronavirus, resulting in a temporary halt of regular healthcare services. In Pakistan, several crucial primary health care services were discontinued during the COVID-19 epidemic, resulting in millions of children being deprived of polio vaccination (32). Uganda and Sierra Leone had similar reductions in vaccination rates, with decreases ranging from 21 to 66% and 18 to 29%, respectively. These implications extend beyond vaccination programs and also include a drop in other healthcare services, such as a fall of 17%−34% in family planning and HIV services in the Democratic Republic of Congo (33).

The COVID-19 pandemic has had a substantial impact on healthcare systems, not just in low- and middle-income nations but also in higher-income regions like as Europe and the United States (30, 32). European countries showed considerable diversity in their approaches to healthcare practices during the COVID-19 pandemic, but overall, they successfully implemented appropriate measures to tackle this problem (34, 35). Smolić et al. (36) discovered that 44.6% of the participants in their study saw disruptions on healthcare services during the pandemic. The interruptions varied from instances of delays to complete refusal of access to healthcare services. Like Europe, the healthcare management in the United States during the COVID-19 pandemic has varied throughout states and towns, with specific locations encountering significant challenges in managing the epidemic (37). While there were no notable changes in some parts of healthcare services, such as emergency care and maternity health, there was a decline in the consumption of several preventative treatments, elective operations, and in-person office visits (38). After implementing widespread COVID-19 vaccination campaigns, which effectively decreased the number of infected individuals and the mortality rate, the strained circumstances in healthcare institutions progressively improved. This circumstance was linked to a decrease in the workload for healthcare personnel and a subsequent resumption of regular healthcare services (39).

Moreover, the distribution of research and development resources has exhibited an imbalanced focus on COVID-19, while certain illnesses, such as recurrent *Salmonella* infections, received much less emphasis and scrutiny (5). It was leading to a postponement in progress related to other infectious diseases (40, 41). The limited availability of resources might unintentionally impact the identification and management of *Salmonella* infections, particularly within healthcare systems already under significant strain. Hence, it serves as a poignant reminder of the urgency of healthcare systems to adapt and address both acute and persistent health challenges. The association between increasing healthcare objectives and the prevalence of *Salmonella* infections is crucial to be recognized for the advancement of comprehensive healthcare systems.

The pandemic has not only disrupted PHC services, but also exposed vulnerabilities in other public sectors, including infrastructures, supply chains, government preparation and actions, human capital, and public health systems. The pandemic had a substantial impact on people's behavior connected to food and the adoption of safety protocols (4, 12). The implementation of improved sanitation practices, such as regular hand washing, maintaining social distance as well as face mask and hand gloves, has had a substantial impact in reducing the spread of foodborne illnesses, including *Salmonella* infections. The resurgence of home cooking has provided individuals with an increased level of control over the safety of their food (11). Subsequently, there has been a rise in the use of food delivery services (42). It requires more attentiveness to guarantee compliance with food safety regulations throughout food production and delivery.

Yet, the COVID-19 pandemic has also shown weaknesses within global food supply networks (43), emphasizing the imperative of establishing robust systems around food safety, traceability systems, and contingency plans (7, 44). *Salmonella*, a proficient bacterium in capitalizing on vulnerabilities within supply chains, enables contamination through these interruptions (45, 46). Humans have exhibited their ability to adapt their habits and employ safety measures considering the global pandemic (47, 48) and persistent issues such as *Salmonella* infection (49). An in-depth awareness of the inherent fluidity in the production and consumption of food is essential for successfully addressing new challenges, such as the COVID-19 pandemic, and for guaranteeing the long-term stability and resilience of the food system.

## Enhancing public health strategies

The COVID-19 pandemic and the persistence of *Salmonella* pose substantial challenges to public health (13, 14). Upon further contemplation, it becomes evident that the pandemic has imparted several valuable lessons, including the urgency of crisis management, the indispensability of resilient healthcare systems, and the imperative nature of international collaboration among nations (47). The preceding lessons have contributed to enhancing the effectiveness of public health systems, reinforcing monitoring efforts, and promoting collaboration in the fight against pathogens such as persistent *Salmonella*. Moreover, it is necessary to emphasize the value of effective communication. The efficient transmission of public health communication plays a pivotal role in promoting preventive behaviors, disseminating accurate details, and countering disinformation (50). Integrating these measures for addressing *Salmonella* might reduce the risk associated with foodborne diseases. For example, the development of telemedicine has become a prominent method of delivering healthcare (51), which is particularly advantageous for infectious diseases. This strategy has demonstrated its ability to enhance healthcare accessibility, particularly in places that lack adequate medical services (52).

Telemedicine has been extensively utilized, particularly in the United States, with its usage significantly expanding during the COVID-19 epidemic (52). This method is particularly beneficial since it allows for the real-time monitoring of patients, which is essential during a pandemic to reduce the danger of pathogens transmission. It also facilitates the provision of remote healthcare assistance and home care services for patients who are in quarantine or isolation. This strategy not only safeguards healthcare practitioners, but also protects patients themselves. Telemedicine is anticipated to have the advantage of reducing hospital admissions and lowering costs for patients. Furthermore, telemedicine facilitates contact between different medical specialties and allows for the efficient collection of data, leading to improved access to healthcare information (51).

In addition to its use in pandemics, telemedicine has the potential to be applied to combating endemic illnesses, as pathogens, including *Salmonella*, may quickly spread across international borders. An all-encompassing and flexible approach is required to address the overlap between COVID-19 and *Salmonella* infections. Sharing information promptly through telemedicine can enhance preparedness for efficiently handling simultaneous health crises. In an era of interrelated risks, understanding the connections between various health conditions is essential for adopting a comprehensive strategy to protect public health.

## Discussions: preparedness for future health crises

The simultaneous presence of *Salmonella* and SARS-CoV-2 infections emphasizes the urgent need for a significant transformation in worldwide health readiness (11). The COVID-19 pandemic has emphasized the need to carefully assess and examine strategies for preventing the spread of infectious diseases, including *Salmonella* infections (53). Thoroughly examining several important conversations and carrying out planned actions is needed. It effectively deals with future health issues, including temporary pandemics and ongoing endemic risks. In addition, the presence of comorbidities of COVID-19 with other infectious disease might also heighten an individual's vulnerability to severe sickness or the acquisition of new microbial illnesses, including the possibility of developing a combined infection with pathogenic bacteria like *Salmonella* (54, 55).

The pandemic has brought to the forefront the vulnerabilities in healthcare systems globally, underscoring the pressing necessity for resilience (56). The inadequacies in the ability to handle sudden increases in capacity, shortages in essential medical resources, and the overwhelming workload on healthcare staff have emphasized the need for a strong and adaptable healthcare system (30, 57). Integrated surveillance systems are essential in pandemics since they enable prompt response measures and efficient allocation of resources (58). The knowledge gained from the monitoring efforts carried out during the COVID-19 pandemic has the capacity to strengthen comprehensive surveillance systems for other infections.

The diagnostic challenges presented by the pandemic require the development of novel analytical methods that are fast, accurate, and easily accessible. Point-of-care testing and telemedicine might enhance diagnostic capacities, especially in low-resource settings (51, 52). The response to the COVID-19 pandemic has demonstrated the significance of global collaboration (47), which could serve as a paradigm for addressing future health emergencies. It entails prioritizing prompt data sharing, coordinating research efforts, and ensuring equitable access to medicines. Community involvement and education are essential for public health, particularly in enhancing community understanding and empowerment concerning cleanliness, vaccination, and appropriate food handling practices. The COVID-19 pandemic has brought attention to the importance of implementing adaptable and rigorous food safety protocols, establishing resilient supply chains, conducting thorough inspections, and educating consumers to mitigate the dangers of foodborne infections, such as salmonellosis and gastroenteritis.

In addition, appropriate treatment protocols to effectively address the infections were of the highest priority. Strict stewardship, careful monitoring of antimicrobial use, and the creation of novel, cutting-edge ways to treat infections are all necessary to avoid any follow-up issues such as antimicrobial resistance. Antimicrobial resistance development, which has also emerged in several strains of *Salmonella*, is recognized as a perpetual burden. It is imperative for healthcare systems to actively participate in scenario planning and readiness drills to identify any gaps in preparedness, formulate appropriate response plans, and establish a well-coordinated approach for handling future emergencies.

In summary, the interrelationship between *Salmonella* and COVID-19 highlights the imperative need for a comprehensive, flexible, and forward-looking plan for global health preparedness. The acquisition of valuable lessons from previous events and the adoption of novel strategies are the most important in safeguarding communities, healthcare systems, and global health security in an era marked by interconnected health risks.

## Author contributions

WP: Conceptualization, Writing—review & editing, Writing—original draft.

## References

[1] Ürkmez FY Atalay T. *Salmonella* bacteremia accompanying COVID-19: the first *Salmonella* co-infection in the world unrelated to Pakistan. Mikrobiyol Bul. (2022) 56:357–64. 10.5578/mb.20229814 35477237

[2] Popa GL Popa MI. *Salmonella* spp. infection-a continuous threat worldwide. Germs. (2021) 11:88–96. 10.18683/germs.2021.1244 33898345 PMC8057844

[3] Pradhan D Negi VD. Stress-induced adaptations in *Salmonella*: a ground for shaping its pathogenesis. Microbiol Res. (2019) 229:126311. 10.1016/j.micres.2019.126311 31446332

[4] Onyeaka H Mazi IM Oladunjoye IO Njoagwuani EI Akegbe H Dolapo OA. Impact of COVID-19 on foodborne illness in Africa – a perspective piece. J Infect Public Health. (2023) 16:651–9. 10.1016/j.jiph.2023.02.018 36917919

[5] Abdul Aziz JM Abdullah SK Al-Ahdal TMA Gubari MIM Rashid MJ Tahir KS. Diagnostic bias during the COVID-19. A rare case report of *Salmonella Typhi*. Ann Med Surg. (2022) 74:103282. 10.1016/j.amsu.2022.103282 35096387 PMC8789390

[6] Aday S Aday MS. Impact of COVID-19 on the Food Supply Chain. Food Quality and Safety. Oxford: Oxford University Press (2020). 10.1093/fqsafe/fyaa024 PMC7499675 40504224

[7] Barman A Das R Kanti De P. Impact of COVID-19 in food supply chain: disruptions and recovery strategy. Curr Res Behav Sci. (2021) 2:100017. 10.1016/j.crbeha.2021.100017 38620642 PMC7987986

[8] Salehi M Nourbakhsh SMK Ardakani MV Abdollahi A Khaki PA Aliramezani A. Bilateral hip septic arthritis caused by nontyphoidal *salmonella* group D in a 16-year-old girl with COVID-19: a case report. Int J Surg Case Rep. (2022) 95:107202. 10.1016/j.ijscr.2022.107202 35661497 PMC9163488

[9] Lin L Jiang X Zhang Z Huang S Zhang Z Fang Z. Gastrointestinal symptoms of 95 cases with SARS-CoV-2 infection. Gut. (2020) 69:997–1001. 10.1136/gutjnl-2020-321013 32241899

[10] Bengoechea JA Bamford CGG. SARS-CoV-2, bacterial co-infections, and AMR: the deadly trio in COVID-19? EMBO Mol Med. (2020) 12:e12560. 10.15252/emmm.202012560 32453917 PMC7283846

[11] Mughini-Gras L Chanamé Pinedo L Pijnacker R van den Beld M Wit B Veldman K. Impact of the COVID-19 pandemic on human salmonellosis in the Netherlands. Epidemiol Infect. (2021) 149:e254. 10.1017/S0950268821002557 36318161 PMC8692841

[12] Davis BP Amin J Franklin N Beggs PJ. Salmonellosis in Australia in 2020: possible impacts of COVID-19 related public health measures. Commun Dis Intell. (2022) 46:1–17. 10.33321/cdi.2022.46.2 35092999

[13] Haqqi A Khurram M Din MSU Aftab MN Ali M Ahmed H. COVID-19 and *Salmonella* Typhi co-epidemics in Pakistan: a real problem. J Med Virol. (2021) 93:184–6. 10.1002/jmv.26293 32648942 PMC7405091

[14] Yogo A Yamamoto S Iwamoto N Aoki K Motobayashi H Tochitani K. Non-typhoidal *Salmonella* Bacteremia in COVID-19 with recrudescence of fever after corticosteroid discontinuation: a case report. IDCases. (2022) 27:e01415. 10.1016/j.idcr.2022.e01415 35096529 PMC8779851

[15] Rawson TM Moore LSP Zhu N Ranganathan N Skolimowska K Gilchrist M. Bacterial and fungal coinfection in individuals with coronavirus: a rapid review to support COVID-19 antimicrobial prescribing. Clin Infect Dis. (2020) 71:2459–68. 10.1093/cid/ciaa530 32358954 PMC7197596

[16] Cheng RA Eade CR Wiedmann M. Embracing diversity: differences in virulence mechanisms, disease severity, and host adaptations contribute to the success of nontyphoidal *Salmonella*as a foodborne pathogen. Front Microbiol. (2019) 10:1368. 10.3389/fmicb.2019.01368 31316476 PMC6611429

[17] Ray LC Collins JP Griffin PM Shah HJ Boyle MM Cieslak PR. Decreased incidence of infections caused by pathogens transmitted commonly through food during the COVID-19 pandemic-foodborne diseases active surveillance network, 10 U.S. sites, 2017-2020. MMWR Morb Mortal Wkly Rep. (2021) 70:1332–36. 10.15585/mmwr.mm7038a4 34555002 PMC8459900

[18] Uzzau S Brown DJ Wallis T Rubino S Leori G Bernard S. Host adapted serotypes of *Salmonella enterica*. Epidemiol Infect. (2000) 125:229–55. 10.1017/S0950268899004379 11117946 PMC2869595

[19] Kozytska T Chechet O Garkavenko T Nedosekov V Haidei O Gorbatiuk O. Antimicrobial resistance of *Salmonella* strains isolated from food products of animal origin in Ukraine between 2018-2021. Ger J Vet Res. (2023) 3:24–30. 10.51585/gjvr.2023.1.0049

[20] Verma S Senger S Cherayil BJ Faherty CS. Spheres of influence: insights into *Salmonella* pathogenesis from intestinal organoids. Microorganisms. (2020) 8:504. 10.3390/microorganisms8040504 32244707 PMC7232497

[21] Ngogo FA Joachim A Abade AM Rumisha SF Mizinduko MM Majigo MV. Factors associated with *Salmonella* infection in patients with gastrointestinal complaints seeking health care at regional hospital in southern highland of Tanzania. BMC Infect Dis. (2020) 20:135. 10.1186/s12879-020-4849-7 32050928 PMC7017463

[22] Bassey EE Hasan MM Costa ACDS Tsagkaris C Aborode AT Karra-Aly A. Typhoid fever and COVID-19 pandemic in Nigeria: a call for coordinated action. Einstein. (2021) 19:eCE679. 10.31744/einstein_journal/2021CE6796 34932777 PMC8687648

[23] Chirwa EB Dale H Gordon MA Ashton PM. What Is the Source of Infections Causing Invasive Nontyphoidal Salmonella Disease? Open Forum Infectious Diseases. Oxford: Oxford University Press (2023). 10.31219/osf.io/5b3un PMC10004642 36910696

[24] Rahman HS Aziz MS Hussein RH Othman HH Salih Omer SH Khalid ES. The transmission modes and sources of COVID-19: a systematic review. Int J Surg Open. (2020) 26:125–36. 10.1016/j.ijso.2020.08.017 34568614 PMC7484735

[25] Aiyegbusi OL Hughes SE Turner G Rivera SC McMullan C Chandan JC. Symptoms, complications and management of long COVID: a review. J R Soc Med. (2021) 114:428–42. 10.1177/01410768211032850 34265229 PMC8450986

[26] Saeed U Uppal SR Piracha ZZ Uppal R. Azithromycin treatment for SARS-CoV-2-related COVID-19 pandemic could worsen extensively drug resistant (Xdr) typhoid: a risk of losing the last bullet against *Salmonella enterica* Serovar Typhi. Jundishapur J Microbiol. (2021) 14:e113874. 10.5812/jjm.113874

[27] Singh M Chakraborty A Tyagi AK. False sero-positivity of *Salmonella* Typhi specific antibody in dengue and corona virus infected patients: an observational study. J Pure Appl Microbiol. (2023) 17:434–38. 10.22207/JPAM.17.1.35

[28] Bogdan I Citu C Bratosin F Malita D Romosan I Gurban CV. The impact of multiplex PCR in diagnosing and managing bacterial infections in COVID-19 patients self-medicated with antibiotics. Antibiotics. (2022) 11:437. 10.3390/antibiotics11040437 35453189 PMC9025156

[29] Carpenter RE Tamrakar V Chahar H Vine T Sharma R. Confirming multiplex RT-QPCR Use in COVID-19 with next-generation sequencing: strategies for epidemiological advantage. Glob Health Epidemiol Genom. (2022) 2022:2270965. 10.1155/2022/2270965 35950011 PMC9339135

[30] Leite H Lindsay C Kumar M. COVID-19 outbreak: implications on healthcare operations. TQM J. (2021) 33:247–56. 10.1108/TQM-05-2020-0111

[31] Pujolar G Oliver-Anglès A Vargas I Vázquez ML. Changes in access to health services during the COVID-19 pandemic: a scoping review. Int J Environ Res Public Health. (2022) 19:1749. 10.3390/ijerph19031749 35162772 PMC8834942

[32] Pradhan NA Samnani AABA Abbas K Rizvi N. Resilience of primary healthcare system across low- and middle-income countries during COVID-19 pandemic: a scoping review. Health Res Policy Syst. (2023) 21:98. 10.1186/s12961-023-01031-4 37723533 PMC10507852

[33] Kasonia K Tindanbil D Kitonsa J Baisley K Zalwango F Enria L. The impact of the COVID-19 pandemic on the provision and utilisation of primary health care services in Goma, Democratic Republic of the Congo, Kambia District, Sierra Leone and Masaka District, Uganda. PLoS ONE. (2023) 18:e0286295. 10.1371/journal.pone.0286295 37267240 PMC10237403

[34] Panteli D Reichebner C Rombey T Berger E Winkelmann J Eckhardt H. Health care patterns and policies in 18 European Countries during the first wave of the COVID-19 pandemic: an observational study. Eur J Public Health. (2022) 32:557–64. 10.1093/eurpub/ckac059 35639951 PMC9341638

[35] Wanat M Hoste M Gobat N Anastasaki M Böhmer F Chlabicz S. Transformation of primary care during the COVID-19 pandemic: experiences of healthcare professionals in eight european countries. Br J Gen Pract. (2021) 71:e634–42. 10.3399/BJGP.2020.1112 33979303 PMC8274627

[36] Smolić Š Cipin I Medimurec P. Access to healthcare for people aged 50+ in europe during the COVID-19 outbreak. Eur J Ageing. (2022) 19:793–809. 10.1007/s10433-021-00631-9 34149338 PMC8195455

[37] O'Reilly-Shah VN Gentry KR Van Cleve W Kendale SM Jabaley CS Long DR. The COVID-19 pandemic highlights shortcomings in US health care informatics infrastructure: a call to action. Anesth Analg. (2020) 131:340–44. 10.1213/ANE.0000000000004945 32366769 PMC7219836

[38] Omer SB Malani P Del Rio C. The COVID-19 pandemic in the US: a clinical update. JAMA. (2020) 323:1767–8. 10.1001/jama.2020.5788 32250388

[39] Mohammed I Nauman A Paul P Ganesan S Chen KH Jalil SMS. The efficacy and effectiveness of the COVID-19 vaccines in reducing infection, severity, hospitalization, and mortality: a systematic review. Hum Vaccines Immunother. (2022) 18:2027160. 10.1080/21645515.2022.2027160 35113777 PMC8862168

[40] AlNaamani K AlSinani S Barkun AN. Medical research during the COVID-19 pandemic. World J Clin Cases. (2020) 8:3156–63. 10.12998/wjcc.v8.i15.3156 32874970 PMC7441262

[41] Fanning JP Murthy S Obonyo NG Baillie JK Webb S Dalton HJ. Global infectious disease research collaborations in crises: building capacity and inclusivity through cooperation. Global Health. (2021) 17:84. 10.1186/s12992-021-00731-2 34311748 PMC8313114

[42] Poon WC En Hui Tung S. The rise of online food delivery culture during the COVID-19 pandemic: an analysis of intention and its associated risk. Eur J Manag Bus Econ. (2022) 33:54–73. 10.1108/EJMBE-04-2021-0128

[43] Swinnen J Vos R. COVID-19 and impacts on global food systems and household welfare: introduction to a special issue. Agric Econ. (2021) 52:365–74. 10.1111/agec.12623 34149127 PMC8206861

[44] Kakaei H Nourmoradi H Bakhtiyari S Jalilian M Mirzaei A. Effect of COVID-19 on food security, hunger, and food crisis. In:Dehghani MH Karri RR Roy S, editors. COVID-19 and the Sustainable Development Goals. Amsterdam: Elsevier (2022). p. 3–29. 10.1016/B978-0-323-91307-2.00005-5

[45] Rajan K Shi Z Ricke SC. Current aspects of *Salmonella* contamination in the US poultry production chain and the potential application of risk strategies in understanding emerging hazards. Crit Rev Microbiol. (2017) 43:370–92. 10.1080/1040841X.2016.1223600 27869522

[46] Sohail MN Rathnamma D Priya SC Isloor S Naryanaswamy HD Ruban SW. *Salmonella* from farm to table: isolation, characterization, and antimicrobial resistance of *Salmonella* from commercial broiler supply chain and its environment. BioMed Res Int. (2021) 2021:3987111. 10.1155/2021/3987111 34660787 PMC8514274

[47] Megahed NA Ghoneim EM. Antivirus-built environment: lessons learned from COVID-19 pandemic. Sustain Cities Soc. (2020) 61:102350. 10.1016/j.scs.2020.102350 32834930 PMC7313520

[48] Kafi A Zainuddin N Saifudin AM Shahron SA Razalli MR Musa S. Meta-analysis of food supply chain: pre, during and post COVID-19 pandemic. Agric Food Secur. (2023) 12:27. 10.1186/s40066-023-00425-5

[49] Mkangara M. Prevention and control of human *Salmonella enterica* infections: an implication in food safety. Int J Food Sci. (2023) 2023:8899596. 10.1155/2023/8899596 37727836 PMC10506869

[50] Porat T Nyrup R Calvo RA Paudyal P Ford E. Public health and risk communication during COVID-19—enhancing psychological needs to promote sustainable behavior change. Front Public Health. (2020) 8:573397. 10.3389/fpubh.2020.573397 33194973 PMC7652763

[51] Haleem A Javaid M Singh RP Suman R. Telemedicine for healthcare: capabilities, features, barriers, and applications. Sens Int. (2021) 2:100117. 10.1016/j.sintl.2021.100117 34806053 PMC8590973

[52] Barbosa W Zhou K Waddell E Myers T Dorsey ER. Improving access to care: telemedicine across medical domains. Annu Rev Public Health. (2021) 42:463–81. 10.1146/annurev-publhealth-090519-093711 33798406

[53] Todd E. Food-borne disease prevention and risk assessment. Int J Environ Res Public Health. (2020) 17:5129. 10.3390/ijerph17145129 32708573 PMC7399861

[54] Jakhmola S Indari O Baral B Kashyap D Varshney N Das A. Comorbidity assessment is essential during COVID-19 treatment. Front Physiol. (2020) 11:984. 10.3389/fphys.2020.00984 32903640 PMC7438844

[55] Jakhmola S Indari O Kashyap D Varshney N Rani A Sonkar C. Recent updates on COVID-19: a holistic review. Heliyon. (2020) 6:e05706. 10.1016/j.heliyon.2020.e05706 33324769 PMC7729279

[56] Haldane V De Foo C Abdalla SM Jung AS Tan M Wu S. Health systems resilience in managing the COVID-19 pandemic: lessons from 28 countries. Nat Med Nat Res. (2021) 27:964–80. 10.1038/s41591-021-01381-y 34002090

[57] Barach P Fisher SD Adams MJ Burstein GR Brophy PD Kuo DZ. Disruption of healthcare: will the COVID pandemic worsen non-COVID outcomes and disease outbreaks? Prog Pediatr Cardiol. (2020) 59:101254. 10.1016/j.ppedcard.2020.101254 32837144 PMC7274978

[58] Lal A Ashworth HC Dada S Hoemeke L Tambo E. Optimizing pandemic preparedness and response through health information systems: lessons learned from ebola to COVID-19. Disaster Med Public Health Prep. (2022) 16:333–40. 10.1017/dmp.2020.361 33004102 PMC7642496

